# Cardiac Damage in Patients Infected with Different SARS-CoV-2 Variants of Concern

**DOI:** 10.3390/microorganisms12122617

**Published:** 2024-12-18

**Authors:** Francesco Robert Burkert, Martina Oberhollenzer, Daniela Kresse, Sarah Niederreiter, Vera Filippi, Lukas Lanser, Günter Weiss, Rosa Bellmann-Weiler

**Affiliations:** Department of Internal Medicine II, Medical University of Innsbruck, Anichstraße 35, 6020 Innsbruck, Austria; francesco.burkert@i-med.ac.at (F.R.B.); martina.oberhollenzer@tirol-kliniken.at (M.O.); daniela.kresse@i-med.ac.at (D.K.); vera.filippi@i-med.ac.at (V.F.); guenter.weiss@i-med.ac.at (G.W.)

**Keywords:** COVID-19, cardiac biomarkers, omicron

## Abstract

Coronavirus Disease 2019 causes significant morbidity, and different variants of concern (VOCs) can impact organ systems differently. We conducted a single-center retrospective cohort analysis comparing biomarkers and clinical outcomes in hospitalized patients infected with the wild-type or Alpha (wt/Alpha) VOC against patients infected with the Omicron VOC. We included 428 patients infected with the wt/Alpha VOC and 117 patients infected with the Omicron VOC. The Omicron cohort had higher maximal median high-sensitivity Troponin-T (hs-TnT) levels (wt/Alpha: 12.8 ng/L, IQR 6.6–29.5 vs. Omicron: 27.8 ng/L, IQR 13.7–54.0; *p* < 0.001) and N-terminal prohormone of brain natriuretic peptide (NT-proBNP) (wt/Alpha: 256 ng/L, IQR 74.5–913.5 vs. Omicron: 825 ng/L, IQR 168–2759; *p* < 0.001) levels. This remained true for patients under 65 years of age and without pre-existing cardiovascular disease (hs-TnT (wt/Alpha: 6.1 ng/L, IQR 2.5–10.25 vs. Omicron: 8.6 ng/L, IQR 6.2–15.7; *p* = 0.007) and NT-proBNP (wt/Alpha: 63 ng/L, IQR 25–223.75 vs. Omicron: 158 ng/L, IQR 75.5–299.5; *p* = 0.006)). In-hospital mortality was similar between the two groups (wt/Alpha: 53 or 12.7% vs. Omicron: 9 or 7.7%; *p* = 0.132) and more patients infected with wt/Alpha VOC required intensive care admission (wt/Alpha: 93 or 22.2% vs. Omicron: 14 or 12%; *p* = 0.014). Increased cardiac biomarkers were correlated with a higher risk of mortality and ICU admission in both groups. Herein, we detected higher levels of cardiac biomarkers in hospitalized patients infected with the Omicron VOC when compared to wt/Alpha, being indicative of higher cardiac involvement. Although hs-TnT and NT-proBNP levels were higher in the Omicron cohort and both markers were linked to in hospital mortality in both groups, the mortality rates were similar.

## 1. Introduction

At the end of 2019, a new strain of coronavirus, Severe Acute Respiratory Distress Syndrome Coronavirus 2 (SARS-CoV-2), emerged in China as a causative agent for Coronavirus Disease 2019 (COVID-19), resulting in a pandemic [1]. Over time, the initial wild-type (wt) virus strain mutated, resulting in the emergence of different variants of concern (VOCs), which presented varying transmissibility, organ involvement and disease severity [2]. Over time, effective antiviral therapies have become available to treat SARS-CoV-2. Remdesivir, a novel nucleotide analog, may reduce mortality and the need for mechanical ventilation in patients with severe disease [3,4]. Nirmatrelvir–ritonavir, a combination of oral protease inhibitors, is a therapeutic option for the outpatient setting which has been proven to reduce symptoms, as well as mortality and hospitalization [5]. Vaccines have also been made widely available, which are effective in reducing disease severity, the need for hospitalization and mortality [6,7,8]. COVID-19 manifests primarily as a respiratory infection; however, other organ systems are also affected, as most cells express the viral binding and entry receptor, Angiotensin-converting enzyme 2 [1]. Specifically, the involvement of the cardiovascular system has been widely reported, ranging from thrombotic complications, such as pulmonary embolism or myocardial infarction, to myocarditis and pericarditis [9]. High-sensitivity Troponin-T (hs-TnT) has become one of the most widely validated biomarkers potentially indicating myocardial damage, alongside other biomarkers such as creatine kinase (CK). The N-terminal prohormone of brain natriuretic peptide (NT-proBNP) is commonly used for the evaluation of cardiac insufficiency [10]. To better understand the importance and magnitude of cardiac involvement in patients infected by different SARS-CoV-2 VOCs in our hospital, we performed this retrospective cohort analysis during the first and third waves of the COVID-19 pandemic, comparing patients infected with the wild-type virus or the Alpha VOC to patients infected with the Omicron VOC.

## 2. Materials and Methods

### 2.1. Study Design and Institutional Review Board Approval

We conducted a retrospective cohort analysis of patients admitted to the Innsbruck university hospital during two separate waves of the COVID-19 pandemic. Since our study was retrospective, informed consent was waived according to the guidelines of our local ethics committee, which approved the data collection (decree number EK-1167/2020).

The inclusion criterion for our study was hospitalization during the study periods because of PCR-confirmed COVID-19. The exclusion criteria were outpatient treatment or complete lack of cardiovascular biomarker measurement during the hospital stay, as well as age under 18 years. The first inclusion period started in January 2020 and ended in February 2021, while the second started in January 2022 and ended in March 2022.

SARS-CoV-2 variant sequencing was not yet performed in our center in the first period of this study; therefore, we assumed that patients were infected with either the wild-type virus or the Alpha VOC (wt/Alpha), which were circulating at the time. Variant sequencing was available in the second period of this study; therefore, we only included patients infected with the Omicron VOC.

### 2.2. Anthropometric and Biochemical Parameters

For patients included in this retrospective analysis, we collected data regarding demographics and pre-existing conditions. We furthermore collected data on the cardiovascular biomarkers hs-TnT, NT-proBNP and CK, as well as the renal biomarker creatinine, which we then used to estimate the glomerular filtration rate (GFR) using the Modification of Diet in Renal Disease (MDRD) formula [11]. Our laboratory’s measurable cutoff for NT-pro-BNP is 50 ng/L, so values under 50 ng/L were approximated to a value of 25 ng/L. The maximal values during hospital stay were used for statistical analysis. Our laboratory’s measurable cutoff for TnT is 5 ng/L, so lower values were approximated to 2.5 ng/L. The maximal values during hospital stay were used for statistical analysis. For high-sensitivity troponin-T, we used the Elecsys^®^ Troponin T-high-sensitivity assay from Roche (Basel, Switzerland), and the cutoff for elevated values was 14 ng/L, as per manufacturer recommendations. We additionally recorded C-reactive protein (CRP) as a surrogate of inflammation. We recorded whether patients required intensive care admission or died during their hospital stay.

We conducted a further subgroup analysis to identify predictors of mortality and need for intensive care.

### 2.3. Statistical Analysis

All data with normal distribution were tested for correlation using one-way analysis of variance (ANOVA), and data with non-normal distribution were analyzed using the Kruskal–Wallis test by ranks. Categorical variables were analyzed by Chi-Square Test or Fisher’s Exact Test, depending on sample size. For pre-existing diseases, the odds ratio was calculated to analyze its correlation with mortality.

Our statistical analyses were performed using R Statistics v.4.3.3 and the packages tableone v.0.13.2 and summarytools v.1.0.1; graphic generation was performed with the package ggplot2 v.3.5.1 and finalfit v.1.0.8 in R Statistics and Microsoft Excel 2019.

## 3. Results

We screened 810 patients and included 418 patients in the first study period. Patients were excluded due to incomplete datasets. All of the included patients in the first study period were assumed to be infected with SARS-CoV-2 wt or Alpha variant. Of these patients, 256 (61.2%) were males and the mean age was 65.4 years. In the second period, we screened 181 patients and included 117; exclusions reasons involved an incomplete dataset and a VOC other than Omicron or missing virus sequencing analysis. Of the 117 included patients, 70 (59.8%) were male and the mean age was 68.6 years (Table 1). The two cohorts were matched based on age, gender, and disease severity, encompassing symptom intensity, complications, and the need for intensive care, since both groups required hospitalization.

Regarding pre-existing conditions, patients admitted with Omicron infections were affected by significantly more cardiovascular diseases (defined as presence or a combination of arterial hypertension, chronic heart failure or coronary heart disease) (wt/Alpha: 241 or 57.9% vs. Omicron: 87 or 74.4%; *p* = 0.001), chronic kidney disease (wt/Alpha: 51 or 12.2% vs. Omicron: 35 or 29.9%; *p* < 0.001) and diabetes (wt/Alpha: 97 or 23.2% vs. Omicron: 40 or 34.2%; *p* = 0.016) when compared to wild-type or Alpha infections. The same was true for a history of tobacco use (Table 1).

**Figure 1:**
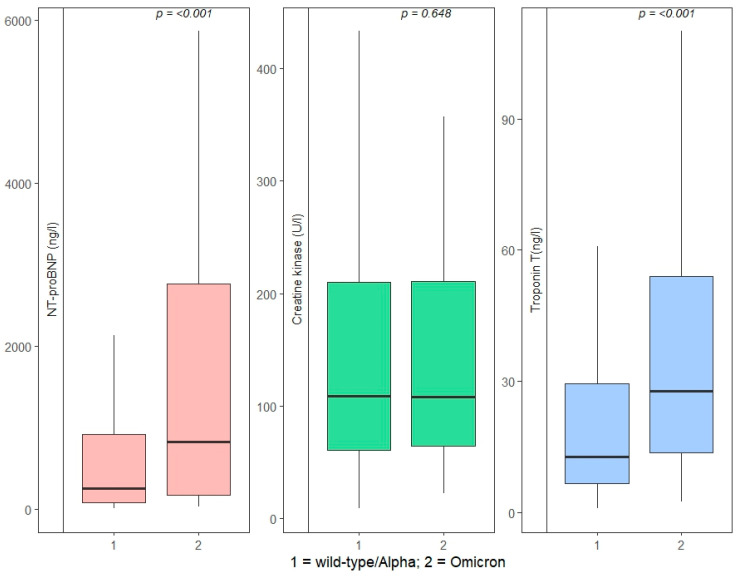
Comparison of NT-proBNP, CK and TnT between wt/Alpha and Omicron (statistical test applied: Kruskal–Wallis test by ranks; statistical significance designated as *p* < 0.05). Interpretation: Lower whisker: lower-quartile group; Box: interquartile range; Horizontal black line: median value; Upper whisker: upper-quartile group; Extreme outliers were excluded to ease interpretation.

A maximum hs-TnT above our laboratory’s cutoff of 14 ng/L was present in 190 (47.3%) patients infected with wt/Alpha and 87 (74.4%) patients infected with the Omicron VoC (*p* < 0.001). The median maximum hs-TnT values were significantly higher in the Omicron VOC cohort (wt/Alpha: 12.8 ng/L, IQR 6.6–29.5 vs. Omicron: 27.8 ng/L, IQR 13.7–54.0; *p* < 0.001), as well as the median NT-proBNP (wt/Alpha: 256 ng/L, IQR 74.5–913.5 vs. Omicron: 825 ng/L, IQR 168–2759; *p* < 0.001), while CK values did not differ significantly (Figure 1).

More patients in the first study period died, but the power of this study was not sufficient to reveal statistical significance (wt/Alpha: 53 or 12.7% vs. Omicron: 9 or 7.7%; *p* = 0.132), although patients hospitalized with Omicron infections had more comorbidities and risk factors for severe courses of infection. More patients infected with wt/Alpha required admission to intensive care units than patients with Omicron (wt/Alpha: 93 or 22.2% vs. Omicron: 14 or 12%; *p* = 0.014).

Higher age correlated with higher mortality in both study periods. Higher levels of maximum CK, hs-TnT, NT-proBNP, CRP and creatinine were also associated with higher mortality (Table 2).

**Figure 2:**
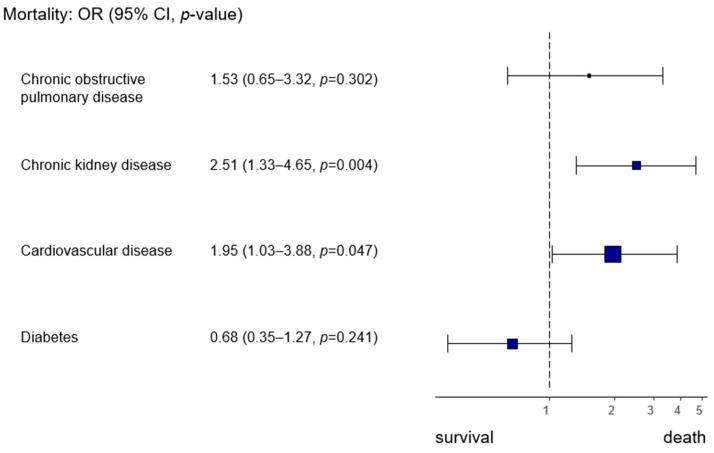
Forest plot analyzing the effects of pre-existing diseases on in-hospital mortality in both study periods.

Pre-existing cardiovascular and renal diseases increased the risk of death during hospitalization (cardiovascular disease OR 1.95, 95% CI 1.03–3.99, *p* = 0.047; chronic kidney disease OR 2.51, 95% CI 1.33–4.65, *p* = 0.004) (Figure 2).

**Figure 3:**
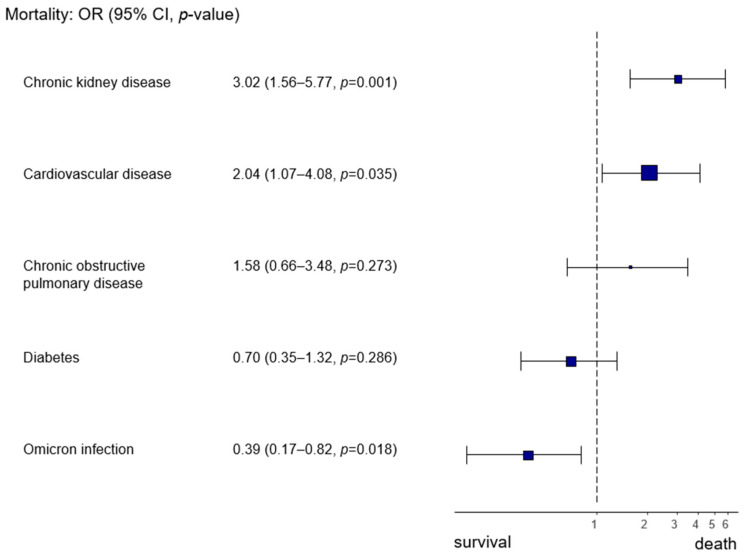
Forest plot analyzing the effects of pre-existing diseases and Omicron infection (as opposed to infection with the wt/Alpha variant) on in-hospital mortality.

By focusing on the Omicron infection as opposed to wt/Alpha infection, we also observed a higher likelihood of survival (Omicron infection OR 0.39, 95% CI 0.17–0.82, *p* = 0.018) (Figure 3).

Of the patients in our study, 107 required admission to an intensive care unit. Factors associated with the need for intensive care were higher age or increased body mass index, as well as higher CK, hs-TnT and CRP. Of our recorded pre-existing conditions, only diabetes correlated with higher risk of ICU admission. (Table 3).

We performed an additional subgroup analysis with patients under 65 years of age or without documented chronic heart disease or coronary heart disease, once again differentiating by VOC. In this subgroup, median hs-TnT remained higher in the Omicron cohort (wt/Alpha: 6.1 ng/L, IQR 2.5–10.25 vs. Omicron: 8.6 ng/L, IQR 6.2–15.7; *p* = 0.007), as well as median NT-proBNP (wt/Alpha: 63 ng/L, IQR 25–223.75 vs. Omicron: 158 ng/L, IQR 75.5–299.5; *p* = 0.006). CK was higher in the wild-type cohort (wt/Alpha: 117 IU/L, IQR 67–192 vs. Omicron: 69 IU/L, IQR 51–118; *p* = 0.027), as was CRP (wt/Alpha: 4.63 mg/dL, IQR 0.99–9.9 vs. Omicron: 1.26 mg/dL, IQR 0.34–4.4; *p* = 0.013) (Table 4).

Since hs-TnT levels are also affected by renal function, we furthermore analyzed creatinine levels. While still within normal range, median creatinine levels were also slightly higher in the Omicron cohort. (wt/Alpha: 0.9 mg/dL, IQR 0.79–1.19 vs. Omicron: 1 mg/dL, IQR 0.84–1.46; *p* = 0.022). A subgroup analysis of patients with an eGFR above the cutoff of 60 mL/min also revealed higher hsTnT values in the Omicron cohort (wt/Alpha: 8.9 ng/L, IQR 5.30–16.55 vs. Omicron: 15.7 ng/L, IQR 7.90–28.75; *p* < 0.001) (Table 5).

An additional subgroup analysis focused on differences in mortality and ICU admission in patients infected with wt/Alpha or Omicron VOC with increased or normal hs-TnT values (cutoff of 14 ng/L), as this marker is associated with acute cardiomyocyte damage. Forty-two (19.8%) patients in the wt/Alpha cohort required ICU admission, despite normal hs-TnT levels, whereas none of the patients infected with the Omicron VOC and a normal hs-TnT had to be admitted to the ICU (Table 6).

For the subgroup of patients with hs-TnT above the 14 ng/L cutoff, rates of ICU admission were not significantly different between the two study periods; however, significantly more among those patients died when infected with wt/Alpha VOC compared to those infected with Omicron VOC (wt/Alpha: 52 (18.8%) vs. Omicron: 8 (9.2%); *p* = 0.006) (Table 7).

## 4. Discussion

COVID-19 emerged in 2020 as a viral infection causing varying severity of illness and affecting different organ systems. In the first months of the pandemic, with the circulating wt and later with the Alpha and Delta variants, pulmonary infections and associated hyper-inflammatory syndromes were the dominating problems, driving the need for hospital admission and potential intensive care unit treatment.

The pulmonary effects of COVID-19 have been well documented, ranging from mild complaints such as cough or sputum production to pneumonia with progression to acute respiratory distress syndrome and respiratory failure [12]. These severe pulmonary manifestations became rare with the rise in the Omicron variants, which appeared to be less pathogenic; at the same time, immune protection emerged as a consequence of vaccination programs and previous infections. SARS-CoV-2 invades target cells after binding to the specific receptor, ACE-2, which is expressed in most organs [1]. Accordingly, patients develop different symptoms of varying severity in specific organ systems including gastro-intestinal, neurological or cardio-vascular problems [13]. As with other viral infections [14], COVID-19 has been shown to increase the incidence of acute cardiac events mainly via inducing inflammation and hypercoagulability [15,16]. SARS-CoV-2 may also cause direct cardiac damage, as evidenced by observations of myocarditis in infected patients, which may be mediated by the pro-inflammatory state induced by the immune response to infection and also by the cytotoxic effects of the virus in tissues. Furthermore, SARS-CoV-2 can invade cardiomyocytes, damaging their contractility as well as dysregulating the renin–angiotensin–aldosterone system and triggering electrical dysfunction [17]. Different SARS-CoV-2 VOCs have been shown to directly affect cardiomyocytes, with the Delta VOC causing the strongest cytopathic effects when compared to the Omicron BA.1 VOC and the early SARS-CoV-2 NL-02-2020 strain [18]. A significant role may also be attributed to microthrombi caused by the hypercoagulable state induced by the infection [19]. Cardiac complications have been described in association with infection with most COVID-19 VOCs [9,20]. In patients infected with the wild-type VOC, more than half of affected patients had pathologic echocardiographic findings such as left/right ventricular abnormalities, with arrhythmias occurring in up to 25% of cases [21,22], and myocarditis or myocardial infarction each occurring in 3% of cases [23]. Data on the Omicron variant continue to emerge, but it appears that the Omicron VOC may more frequently cause cardiovascular complications in younger and white patients, while still having a lower mortality than infections with the wild-type and delta VOCs [20]. A case report documented an instance of myocarditis with evidence of interstitial infiltration of the myocardium by CD3+-T lymphocytes and CD68+-macrophages [24]. The Omicron VOC has been considered to cause milder infections than the previous variants, especially the Delta VOC; however, Omicron has been shown to still cause significant morbidity and mortality compared to other respiratory infections [25]. In our study, we detected significantly higher levels of biomarkers for acute cardiac injury as well as chronic heart stress in patients affected by the Omicron VOC when compared to those affected by the wt/Alpha VOC, indicating that the burden of cardiac damage in Omicron infections should not be underestimated.

Our data furthermore show that Omicron may have more tropism for the heart independent of inflammation, as CRP was higher in younger patients affected by wt/Alpha, while those affected by Omicron more frequently had pathologically increased cardiac biomarkers. A recent study also confirmed that the Omicron BA.2 VOC efficiently infected and injured cardiomyocytes [26].

A correlation between higher cardiac or inflammatory biomarkers and mortality has been hitherto abundantly described and was corroborated by our analysis [27,28,29,30]. The same holds true for admission with serious pre-existing medical conditions such as chronic kidney disease and cardiovascular disease [31]. It is interesting that, while the Omicron cohort presented with significantly more serious pre-existing medical conditions, they did not experience higher mortality, potentially indicating that wt/Alpha infections caused increased disease severity leading to enhanced mortality. The higher presence of comorbidities in the Omicron cohort is potentially also due to the fact that patients with more risk factors such as pre-existing diseases, including cardio-vascular disease or chronic kidney disease, more frequently required hospitalization. When including the type of infection in the mortality analysis with pre-existing diseases, the Omicron infection was associated with reduced mortality when compared to subjects with COVID-19 due to the wt/Alpha VOC. The largest limitation of our study is the direct comparison of clinical outcomes in the two VOCs, since additional antiviral therapeutic options became available during our study. Remdesivir was approved by the European Medical Agency for hospitalized patients in June 2020 [32], and may therefore have been administered in both of our study cohorts. Nirmatrelvir–ritonavir was approved by the European Medical Agency for outpatients in January 2022 [33] and, while not being widely utilized in admitted patients, may have reduced the burden of hospitalization or ICU admission in our region. While the evidence for these antiviral therapies is limited, both treatments may have an impact on mortality, the need for mechanical ventilation, and ICU admission [3,4,5]. Novel vaccines have also been available in Austria since December 2020, and therefore patients in both cohorts may have been vaccinated [6]. More patients in the second cohort may have been vaccinated, as the vaccine was available for the entirety of the second study period, but only two months of the first study period. These vaccines are effective in preventing severe COVID-19, reducing hospital admissions and the need for intensive care, as well as decreasing COVID-19-associated mortality [7,8,34]. Therefore, the introduction of these treatments and vaccines may introduce an additional unmeasured confounder in our statistical analysis of clinical outcomes. We acknowledge this limitation, but still believe in the solidity of our biochemical data. Future studies are warranted to analyze the additional effect of antiviral therapies on cardiac biomarkers, as well as clinical outcomes in patients infected by SARS-CoV-2.

As an additional limitation, ours was a single-center study; therefore, our data may be influenced by local effects and limited patient heterogeneity. A number of patients had to be excluded due to unavailable data regarding the VOC, thereby reducing the statistical power of this study. We did not have data available for all patients with regard to acute cardiac events during their hospital stay; it is therefore not possible to correlate the troponin levels to cardiac outcomes. At least a part of the elevated troponin T levels in the Omicron cohort of our study can be attributed to the higher prevalence of chronic kidney disease within this cohort. However, the troponin T levels remain higher in this cohort even in the subgroup analysis of younger patients without chronic kidney disease, as well as in the subgroup analysis of patients with an eGFR above 60 mL/min. Furthermore, at least for the very early days of the pandemic, a lack of knowledge and fear of a new, unknown infection, as well as the hope of containing the spread of the pandemic, may have led to an increased ease of admission for patients with wt infections when compared to Omicron.

Additional unmeasured confounders may have skewed the results of our study, such as undetected shifting of the demographics of hospitalized patients during the study periods. This may have led to patients with cardiac pathologies or acute cardiac events being under-represented in the first study period or over-represented in the second study period [35].

Despite these limitations, we believe our study provides important and clinically relevant information in showing that cardiac involvement is frequently observed in subjects with viral infection caused by less pathogenic SARS-CoV-2 VOCs, also suggesting that the Omicron variant may possess a higher cardiotropism. Further research is warranted to fully understand the tropism of these viruses for cardiomyocytes and the associated potential increase in morbidity and mortality, as well as the pathogenic mechanisms underlying COVID-19-associated cardiac injury. This may also help to set up preventive strategies that may help to reduce cardiovascular events in patients with COVID-19 or other respiratory viral infections.

## Figures and Tables

**Table 1:** Baseline characteristics of patients with wild-type/Alpha or Omicron infections.

	Overall	wt/Alpha	Omicron	*p*
Number of patients	535	418	117	
Patient age (mean (SD))	66.07 (17.66)	65.29 (17.44)	68.60 (18.07)	0.079
Patient sex = male, *n* (%)	362 (60)	256 (61.2)	70 (59.8)	0.782
Creatine kinase, U/L (median [IQR])	108.00 [62, 211]	108.50 [60.75, 210]	108.00 [64, 211]	0.648
Hs-TnT, ng/L (median [IQR])	15.50 [7.15, 34]	12.85 [6.60, 29.55]	27.80 [13.70, 54]	<0.001
Hs-TnT above 14 ng/L cutoff, *n* (%)	277 (53.4)	190 (47.3)	87 (74.4)	<0.001
NT-proBNP, ng/L (median [IQR])	319.00 [89.75, 1291.75]	256.00 [74.50, 913.5]	825.00 [168, 2759]	<0.001
CRP, mg/dL (median [IQR])	4.86 [1.55, 10.96]	5.08 [1.60, 10.61]	4.37 [1.03, 11.86]	0.498
Creatinine, mg/dL (median [IQR])	0.98 [0.79, 1.27]	0.95 [0.79, 1.19]	1.01 [0.84, 1.46]	0.022
eGFR mL/min (mean (SD))	70.39 (29.04)	72.26 (28.85)	63.72 (28.84)	0.005
In-hospital mortality, *n* (%)	62 (11.6)	53 (12.7)	9 (7.7)	0.132
ICU admission, *n* (%)	107 (20)	93 (22.2)	14 (12)	0.014
Cardiovascular disease, *n* (%)	328 (61.5)	241 (57.9)	87 (74.4)	0.001
Chronic kidney disease, *n* (%)	86 (16.1)	51 (12.2)	35 (29.9)	<0.001
Diabetes, *n* (%)	137 (25.6)	97 (23.2)	40 (34.2)	0.016
Tobacco use, *n* (%)	104 (19.6)	68 (16.4)	36 (31.0)	<0.001

*p* < 0.05 was considered statistically significant; Statistical tests: ANOVA/Kruskal–Wallis test by ranks/Chi-Square Test/Fisher’s Exact Test.

**Table 2:** Factors associated with in-hospital mortality in all patients independent of the VOC.

	Overall	Survival	In-Hospital Mortality	*p*
Number of patients	535	471	62	
Patient age (mean (SD))	66.07 (17.66)	64.28 (17.62)	80.02 (10.44)	<0.001
Patient sex = male, *n* (%)	326 (60.9)	282 (59.9)	43 (69.4)	0.15
Creatine kinase, U/L (median [IQR])	108.00 [62.00, 211.00]	107.00 [62.00, 194.00]	178.50 [63.25, 519.50]	0.005
NT-proBNP (median [IQR])	319.00 [89.75, 1291.75]	247.00 [78.75, 1148.75]	1076.50 [562.75, 4417.00]	<0.001
Hs-TnT, ng/L (median [IQR])	15.50 [7.15, 34.00]	13.50 [6.65, 29.75]	35.80 [23.35, 112.40]	<0.001
CRP, mg/dL (median [IQR])	4.86 [1.55, 10.96]	4.53 [1.30, 9.90]	9.57 [4.36, 19.70]	<0.001
Creatinine, mg/dL (median [IQR])	0.98 [0.79, 1.27]	0.94 [0.78, 1.17]	1.29 [1.00, 1.98]	<0.001
eGFR mL/min (mean (SD))	70.39 (29.04)	73.30 (28.04)	47.92 (26.45)	<0.001

*p* < 0.05 was considered statistically significant. Statistical tests: ANOVA/Kruskal–Wallis test by ranks/Chi-Square Test/Fisher’s Exact Test.

**Table 3:** Intensive care admission analysis for both study periods.

	Overall	Non-ICU	ICU	*p*
Number of patients	535	428	107	
Patient age (mean (SD))	66.07 (17.66)	66.55 (18.83)	64.14 (11.69)	0.207
Patient sex = male, *n* (%)	326 (60.9)	252 (58.9)	74 (69.2)	0.051
Body mass index (kg/m^2^)	27.01 (5.75)	26.64 (5.38)	28.66 (6.95)	0.003
Creatine kinase, U/L (median [IQR])	108.00 [62.00, 211.00]	105.00 [61.00, 192.00]	146.50 [69.25, 446.75]	0.004
NT-proBNP (median [IQR])	319.00 [89.75, 1291.75]	277.00 [79.00, 1215.00]	420.00 [163.50, 1551.0]	0.051
Hs-TnT, ng/L (median [IQR])	15.50 [7.15, 34.00]	15.00 [6.80, 33.20]	16.75 [10.20, 40.48]	0.036
CRP, mg/dL (median [IQR])	4.86 [1.55, 10.96]	4.03 [1.09, 8.73]	11.10 [5.30, 19.28]	<0.001
Creatinine, mg/dL (median [IQR])	0.98 [0.79, 1.27]	0.95 [0.79, 1.24]	1.05 [0.81, 1.35]	0.127
eGFR mL/min (mean (SD))	70.39 (29.04)	70.69 (29.50)	69.21 (27.21)	0.637
Diabetes, *n* (%)	137 (25.6)	94 (22.0)	43 (40.2)	<0.001

*p* < 0.05 was considered statistically significant. Statistical tests: ANOVA/Kruskal–Wallis test by ranks/Chi-Square Test/Fisher’s Exact Test.

**Table 4:** Subgroup analysis for hospitalized patients under 65 years and without a known cardiovascular disease.

	Overall	wt/Alpha	Omicron	*p*
Number of patients	199	170	29	
Patient age (mean (SD))	48.14 (12.36)	48.16 (12.26)	48.00 (13.15)	0.949
Patient sex = male, *n* (%)	122 (61.3)	109 (64.1)	13 (44.8)	0.049
Body mass index (kg/m^2^)	27.58 (5.76)	28.05 (5.70)	25.19 (5.53)	0.016
Creatine kinase, U/L (median [IQR])	107.50 [62.25, 185.00]	117.00 [67.00, 192.00]	69.00 [51.00, 118.00]	0.027
NT-proBNP (median [IQR])	74.00 [25.00, 235.50]	63.00 [25.00, 223.75]	158.00 [75.50, 299.50]	0.006
Hs-TnT, ng/L (median [IQR])	6.50 [2.50, 10.90]	6.10 [2.50, 10.25]	8.60 [6.20, 15.70]	0.007
CRP, mg/dL (median [IQR])	3.92 [0.79, 9.65]	4.63 [0.99, 9.90]	1.26 [0.34, 4.40]	0.013
Creatinine, mg/dL (median [IQR])	0.88 [0.76, 1.01]	0.87 [0.76, 1.00]	0.89 [0.78, 1.07]	0.473
eGFR mL/min (mean (SD))	90.04 (25.38)	91.60 (23.57)	80.86 (33.19)	0.035
ICU admission, *n* (%)	47 (23.6)	44 (25.9)	3 (10.3)	0.069

*p* < 0.05 was considered statistically significant. Statistical tests: ANOVA/Kruskal–Wallis test by ranks/Chi-Square Test/Fisher’s Exact Test.

**Table 5:** Subgroup analysis for patients with normal renal function (eGFR > 60 mL/min), according to VOC.

	Overall	wt/Alpha	Omicron	*p*
Number of patients	354	287	67	
Patient age (mean (SD))	60.70 (17.61)	60.24 (17.30)	62.69 (18.86)	0.306
Patient sex = male, *n* (%)	214 (60.5)	175 (61.0)	39 (58.2)	0.677
Body mass index (kg/m^2^)	27.06 (5.60)	27.18 (5.68)	26.62 (5.28)	0.477
Creatine kinase, U/L (median [IQR])	99.00 [56.50, 166.00]	97.00 [55.75, 164.25]	100.00 [61.50, 210.00]	0.283
NT-proBNP (median [IQR])	173.00 [25.00, 464.00]	156.00 [25.00, 403.00]	222.00 [130.00, 686.00]	0.009
Hs-TnT, ng/L (median [IQR])	9.70 [5.70, 18.30]	8.90 [5.30, 16.55]	15.70 [7.90, 28.75]	<0.001
CRP, mg/dL (median [IQR])	4.26 [1.08, 9.10]	4.39 [1.38, 9.09]	3.33 [0.76, 9.29]	0.268
In-hospital mortality, *n* (%)	21 (5.9)	17 (5.9)	4 (6.0)	0.994
ICU admission, *n*(%)	66 (18.6)	60 (20.9)	6 (9.0)	0.024

*p* < 0.05 was considered statistically significant. Statistical tests: ANOVA/Kruskal–Wallis test by ranks/Chi-Square Test/Fisher’s Exact Test.

**Table 6:** Subgroup analysis of in-hospital mortality and ICU admission in patients with normal hs-TnT (<14 ng/L).

	Overall	wt/Alpha	Omicron	*p*
Number of patients	258	212	30	
In-hospital mortality, *n* (%)	6 (2.5)	5 (2.4)	1 (3.3)	0.755
ICU admission, *n* (%)	42 (17.4)	42 (19.8)	0 (0.0)	0.007

*p* < 0.05 was considered statistically significant. Statistical tests: Chi-Square Test.

**Table 7:** Subgroup analysis of in-hospital mortality and ICU admission in patients with Hs-TnT ≥ 14 ng/L, compared by infection.

	Overall	wt/Alpha	Omicron	*p*
Number of patients	293	190	87	
In-hospital mortality, *n* (%)	52 (18.8)	44 (23.2)	8 (9.2)	0.006
ICU admission, *n* (%)	64 (23.1)	50 (26.3)	14 (16.1)	0.061

*p* < 0.05 was considered statistically significant. Statistical tests: Chi-Square Test.

## Data Availability

The data presented in this study are available on request from the corresponding author.
